# Testing the metacognitive model against the benchmark CBT model of social anxiety disorder: Is it time to move beyond cognition?

**DOI:** 10.1371/journal.pone.0177109

**Published:** 2017-05-04

**Authors:** Henrik Nordahl, Adrian Wells

**Affiliations:** 1 Department of Psychology, Norwegian University of Science and Technology, Trondheim, Norway; 2 St. Olavs Hospital, Division of Psychiatry, Trondheim, Norway; 3 Division of Psychology and Mental Health, School of Health Sciences, Faculty of Biology, Medicine and Health, University of Manchester, Manchester Academic Health Science Centre, Manchester, United Kingdom; 4 Greater Manchester Mental Health NHS Foundation Trust, Prestwich, United Kingdom; Chiba University Graduate School of Medicine, JAPAN

## Abstract

The recommended treatment for Social Phobia is individual Cognitive-Behavioural Therapy (CBT). CBT-treatments emphasize social self-beliefs (schemas) as the core underlying factor for maladaptive self-processing and social anxiety symptoms. However, the need for such beliefs in models of psychopathology has recently been questioned. Specifically, the metacognitive model of psychological disorders asserts that particular beliefs about thinking (metacognitive beliefs) are involved in most disorders, including social anxiety, and are a more important factor underlying pathology. Comparing the relative importance of these disparate underlying belief systems has the potential to advance conceptualization and treatment for SAD. In the cognitive model, unhelpful self-regulatory processes (self-attention and safety behaviours) arise from (e.g. correlate with) cognitive beliefs (schemas) whilst the metacognitive model proposes that such processes arise from metacognitive beliefs. In the present study we therefore set out to evaluate the absolute and relative fit of the cognitive and metacognitive models in a longitudinal data-set, using structural equation modelling. Five-hundred and five (505) participants completed a battery of self-report questionnaires at two time points approximately 8 weeks apart. We found that both models fitted the data, but that the metacognitive model was a better fit to the data than the cognitive model. Further, a specified metacognitive model, emphasising negative metacognitive beliefs about the uncontrollability and danger of thoughts and cognitive confidence improved the model fit further and was significantly better than the cognitive model. It would seem that advances in understanding and treating social anxiety could benefit from moving to a full metacognitive theory that includes negative metacognitive beliefs about the uncontrollability and danger of thoughts, and judgements of cognitive confidence. These findings challenge a core assumption of the cognitive model and treatment of social phobia and offer further support to the metacognitive model.

## Introduction

There are two main cognitive models and treatments of Social phobia or Social anxiety disorder (SAD) which are recommended by the National Institute for Health and Clinical Excellence [1]; the model by Clark and Wells [2] and the model by Rapee and Heimberg [3]. In support of the guidelines, a recent systematic review and network meta-analysis showed that individual cognitive therapy is more effective than other psychological treatments and drug treatments. In particular, the treatment based on the Clark and Wells [2] model is highly effective [4].

Drawing on the work of Beck and colleagues [5–6] cognitive models assert that maladaptive self-beliefs and assumptions (e.g. “I am foolish”) give rise to negative interpretations of experience, negative feelings, and counter-productive safety behaviours aimed at preventing failure and embarrassment, and they play a key role in the development and maintenance of social anxiety.

In their model of SAD, Clark and Wells [2] assigned a role to schemas or cognitive beliefs, but they also drew on the metacognitive model [7–8] and described how self-regulatory cognitive processes (e.g. self-focused attention) maintained social anxiety. According to Clark and Wells, negative cognitive beliefs (e.g. “I’m boring”) are activated when the person with social phobia enters social situations. This in turn leads to negative interpretations of performance and a shift in attention to self-focus on a biased and distorted inner image of the self. Safety behaviours such as avoiding eye contact or rehearsing sentences in the mind in an attempt to be interesting are used to deal with negative beliefs about how one appears to others but they impair performance and increase self-focused attention. In addition to these factors, anticipatory worry and post-event rumination before and after social encounters contribute to problem maintenance. This pattern of processing can be traced back to underlying negative beliefs and assumptions about the social self.

Rapee and Heimberg’s cognitive-behavioural model [3] shares many features of the Clark and Wells model [2], but in addition argues that social phobic individuals are characterized by maladaptive self-related processing that could also be external, such as scanning the environment for signs of negative evaluation. However, this model also emphasizes cognitive beliefs as the underlying mechanism of all negative self-processing.

There is substantial empirical evidence for the role of self-focused attention and the use of safety behaviours as maintenance factors in social anxiety [9–10]. There are also several studies supporting a role of cognitive beliefs [11]. However, the need for these beliefs or cognitive schemas in explaining the cause of psychopathology has been questioned [7] and Metacognitive therapy [12] which does not deal with such beliefs is proving to be an innovative and highly effective treatment that may be more effective than CBT [13].

More specifically, the Wells and Matthews metacognitive model [7–8, 12] specifies that a different set of beliefs is important and have been overlooked in CBT. The beliefs concerned are metacognitive in nature representing beliefs about thinking: e.g. “When I start worrying I cannot stop” and “I do not trust my memory”). Wells and Matthews [7–8] proposed that metacognitive beliefs are involved in most disorders including social anxiety. For example, the belief that worrying is uncontrollable leads to a persistence of worrying about the social self (e.g. “I’m boring”) because the person does not use their mind to interrupt the process. In the metacognitive model, controlling attention and regulating excessive thinking such as worry are the most important factors underlying pathology, and these processes are directly linked to underlying metacognitive beliefs, and not cognitive beliefs or schemas as emphasised in cognitive models. In this approach, psychological disorder results from a thinking style called the cognitive attentional syndrome (CAS) [12]. The CAS consists of over-thinking in the form of worry/rumination, self-focused attention and maladaptive coping strategies, and this process is seen as the cause of disorder rather than cognitive beliefs or the content of negative appraisals [14].

Thus, the cognitive model is one in which unhelpful self-regulatory processes such as self-attention, worry and safety behaviours arise from cognitive beliefs (schemas; e.g. “I’m boring”) whilst the metacognitive model proposes that such processes arise from metacognitive beliefs (e.g. “I cannot stop worrying about being boring”). In support of the metacognitive model, findings from several studies suggest that metacognitive beliefs play a role in social anxiety [15–17].

Comparing the relative importance of the disparate underlying belief systems in the cognitive versus the metacognitive models has the potential to advance our understanding and treatment and is therefore an important research area. In the present study, we aimed to progress the field by evaluating the absolute and relative fit of the cognitive and metacognitive models. Our research question was: Do these models fit the data and does one model fit better than the other? In the cognitive model the relationship between beliefs and anxiety is both indirect via self-processing and also direct. In the metacognitive model the direct path is possible but not mandatory. However, we retained the direct paths in both models so that the models were comparable and only one parameter (type of belief) was varied. In order to address our research question, we used a longitudinal design with two measuring points and structural equation modelling to test the fit of the cognitive- and the metacognitive model of social anxiety symptoms.

## Methods

### Participants and procedure

The study was conducted from mid-August 2016 in Norway and was approved by the Regional committee for medical and health research ethics (REC; ref.nr. 2016/705). Participants were invited to an online survey on social anxiety through advertisement on social media, and were informed that everybody who completed the survey at both time points would participate in a lottery to win an iPad. Several voluntary organizations for mental health in Norway shared information about the survey with their members and social media followers. In accordance with the ethical approval from REC, informed consent was obtained online following reading of the survey information sheet that was presented on the first page after accessing the link to the survey. No one could access the survey without reading the information sheet and consenting to participate.

A total of 712 participants completed the survey at time 1 (t1), 582 (81.7%) were female, and the mean age was 30.50 (10.38). The second round of questionnaires was administered approximately 8 weeks after completion of the first round. Five-hundred and five (505) participants also completed the questionnaires at time 2 (t2). In this sample, 412 (81.6%) were female, and the mean age was 30.27 (10.17). The drop-out rate from t1 to t2 was therefore 29%.

### Measures

The Social Interaction Anxiety Scale (SIAS) [18] is at 20-item scale that measures fear of and responses to social interactions. It has shown high internal consistency (α = .93) and test-retest reliability (.92) [18]. SIAS has a range from 0 to 80, high scores indicating higher levels of social anxiety. In this study, the Cronbach’s alpha was .95.

The Social Avoidance and Distress scale (SAD) [19] is a 28-item measure of distress in social situations and avoidance, using a true-false scale. Its internal consistency has been found excellent (α = .94) and its test-retest reliability ranged from .68 to .79 [19]. SAD has a range from 0 to 28, high scores indicating higher levels of social anxiety. In this study, the Cronbach’s alpha was .95.

The Social Phobia Rating Scale [20] has five rating-scales assessing key components of the cognitive model and therapy [2]; distress, avoidance, self-consciousness, use of safety behaviours, and negative cognitive beliefs. In the current study, we used the following subscales; Self-consciousness: participants are asked to rate how self-conscious they have felt in social situations the last week on a scale ranging from 0 (not at all) to 8 (extremely). Use of safety behaviours: participants are asked how often they use different examples of safety behaviours when they are socially anxious. Participants give a rating for 15 different examples of safety behaviours, e.g. “try to relax” and “avoid eye contact”, on a scale from 0 (not at all) to 8 (all the time). A total score can be derived by summating the ratings for each item. In the current study, the scale had high internal consistency (α = .88). Cognitive beliefs: participants are asked to rate how much they believe 14 different negative beliefs characterizing social phobia on a scale from 0 (not at all) to 100 (totally convinced that the belief is true) when they are socially anxious, e.g. “I look bad” and “They will notice I’m anxious”. A total score can be derived by summating the belief ratings for each item, so the total scale ranges from 0 to 1400. In the current study, the scale had excellent internal consistency (α = .96).

The Social Imagery Perspective Scale (SIPS: Wells, S1 File) is a 3 item self-report scale measuring the extent people experience a mental image of themselves from an observer-perspective in social situations, e.g. “When I’m in a social situation: I have an inner impression of how I look”. Responses are required on a four-point scale ranging from 0 (never) to 3 (almost always). Prior to inclusion of the SIPS in the present study, we validated the measure in a cross-sectional study of 405 undergraduate students at the Norwegian University of Science and Technology. In this study, the internal consistency of the SIPS was good (α = .78), and it correlated significantly with the self-consciousness scale of the SPRS (r = .35, p < .001). We also evaluated incremental validity, correlating SIPS with SIAS while controlling for self-consciousness as measured by the SPRS, and the incremental predictive validity of the scale was confirmed (r = .19, p < .001). In the present study, the internal consistency of the scale was good (α = .82) and it correlated significantly with the self-consciousness scale of the SPRS (r = .48, p < .001). The incremental predictive validity of the scale was confirmed by correlating SIPS with SIAS while controlling for self-consciousness as measured by the SPRS (r = .29, p < .001).

The Metacognitions questionnaire 30 (MCQ-30) [21] is a 30-item self-report scale measuring metacognitive beliefs and processes about thinking. Responses are required on a four-point scale ranging from 1 (do not agree) to 4 (agree very much), and each subscale has a range from 6–24 points. The MCQ-30 has a five-factor structure: 1) positive beliefs about worry concerning beliefs about the usefulness of worry such as “worrying helps me cope”; 2) negative beliefs concerning the uncontrollability of worry and corresponding danger such as: “worry is uncontrollable” and “I could make myself sick with worrying”; 3) cognitive confidence, which concerns judgements of confidence in memory such as “I do not trust my memory”; 4) need to control thoughts that measures beliefs about the need for mental control such as: “I should be in control of my thoughts all of the time”; and 5) cognitive self-consciousness concerning the tendency to focus on thoughts: e.g. “I monitor my thoughts”. High scores reflect greater dysfunction with the item in question. The measure has shown good internal consistency with Cronbach’s alpha ranging from .72 to .93 [21]. In the current study, the Cronbach’s alpha was .85 for positive metacognitive beliefs, .88 for negative metacognitive beliefs, .89 for cognitive confidence, .83 for need for control, and .78 for cognitive self-consciousness.

## Results

Initially, before testing models we ran correlational analyses to examine the basic pattern of relationships between measures of social anxiety, self-processing, cognitive beliefs and metacognitive beliefs. Table 1 presents descriptive statistics and Pearson product-moment correlations between measures. All of the correlations were positive and significant. The data showed that schemas (SPRS cb) and metacognitions (MCQ-30) measured at time 1 were significant positive predictors of social anxiety measures (SIAS, SAD) at time 2. Cognitive beliefs and metacognitive beliefs measured at t1 were positively inter-correlated.

**Table 1:** Descriptive statistics and bivariate correlations among variables; symptom- (SIAS, SAD) and self-processing variables (SIPS, SPRS self-consciousness, SPRS safety behaviours) from t2, and knowledge structures (SPRS cognitive beliefs, and MCQ-30 metacognitive beliefs) from t1 (n = 505).

		2	3	4	5	6	7	M	SD
**1.**	SIAS_t2	.875*	.503*	.626*	.711*	.805*	.642*	26.74	15.13
**2.**	SAD_t2		.451*	.633*	.687*	.738*	.613*	15.02	8.93
**3.**	SIPS_t2			.420*	.498*	.526*	.480*	4.96	2.37
**4.**	SPRS: sc_t2				.562*	.616*	.547*	3.16	2.65
**5.**	SPRS: sb_t2					.703*	.623*	41.93	24.75
**6.**	SPRS: cb_t1						.697*	684.53	420.21
**7.**	MCQ-30_t1							60.28	16.57

Note: SIAS = Social Interaction Anxiety Scale, SAD = Social Avoidance and Distress scale, SIPS = Social Imagery Perspective Image Scale, SPRS = Social Phobia Rating Scale (sc = self-consciousness, sb = safety behaviours, cb = cognitive beliefs), MCQ-30 = Metacognitions questionnaire 30.

*p < .01

Subsequently, in order to test the fit of the longitudinal cognitive- and the metacognitive models, we used structural equation modelling [22]. Evaluation of model fit was conducted according to Hu and Bentler [23], where the Comparative Fit Index (CFI) and the Tucker-Lewis Index (TLI) should be close to or more than .95, and where a standardized root mean square residual (SRMR) should be less than .08, and a root mean square error of approximation (RMSEA) less than .06, to represent good model fit. We also included the Akaike Information Criterion (AIC) [24–25] where, in the comparison of non-nested models, the model with the minimum AIC value is regarded as the best fitting model.

**Fig 1:**
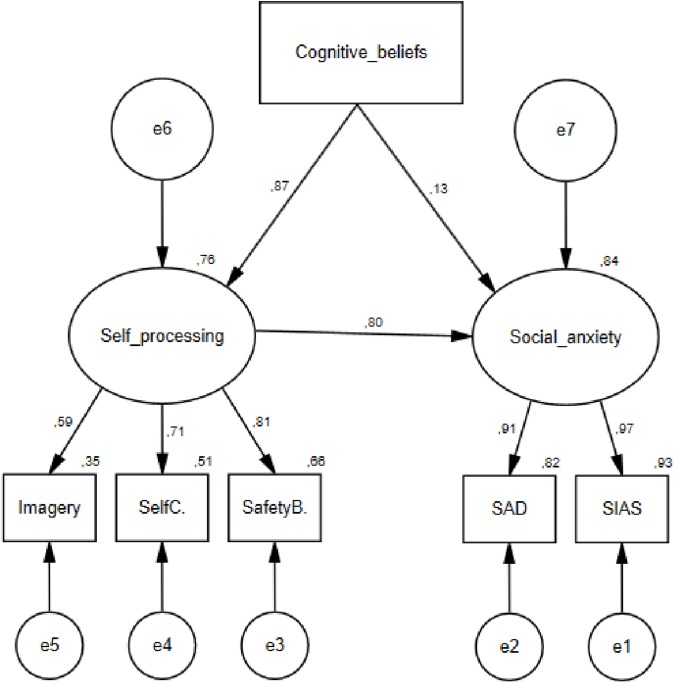
Structure and standardized estimates of the cognitive model of social anxiety.

The cognitive model is specified in Fig 1. Social phobic cognitive beliefs (schemas) were treated as an observed variable and self-processing and social anxiety were latent variables with the observed constructs as indicated in the Figure. We used self-processing variables and social anxiety variables from the t2 dataset (maintenance factors), and cognitive beliefs from the t1 dataset in order to assess causality. The data did not fit the cognitive model well as evidenced by a significant chi-square statistic χ^2^(7) = 20.814, p < .01, however the other fit indices were mixed suggesting a reasonable but not exemplary fit; CFI = .994, TLI = .986, RMSEA = .063, SRMR = .015. The AIC value for the model was 48.814, and the squared multiple correlations were .76 for self-processing, and .84 for social anxiety. All the standardized regression weights in the model except from the path from cognitive beliefs to social anxiety were significant at the .001 level.

**Fig 2:**
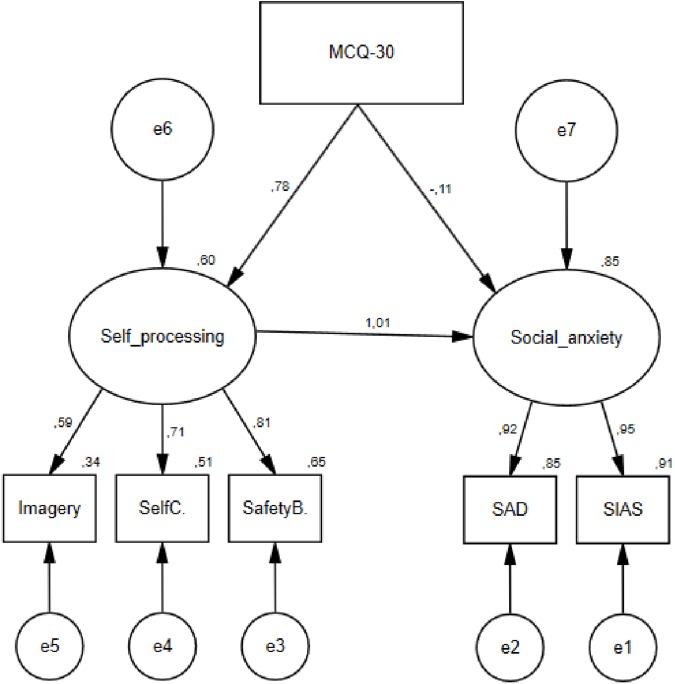
Structure and standardized estimates of the basic metacognitive model of social anxiety.

To test the fit of the metacognitive model, we replaced the cognitive beliefs with the total score of the MCQ-30, but the structure of the rest of the model was the same (Fig 2) and all other parameters were maintained. The fit of the metacognitive model was good χ^2^(7) = 13.269, p = .066, as demonstrated by a non-significant chi-square statistic and good all round fit indices: CFI = .997, TLI = .993, RMSEA = .042, SRMR = .015. The AIC value for the model was 41.269, and the squared multiple correlations were .60 for self-processing, and .85 for social anxiety. All the standardized regression weights in the model except for the path from metacognitive beliefs to social anxiety were significant at the .001 level.

As the metacognitive model showed a good fit and a better fit than the cognitive model, we further explored which domains of metacognitive beliefs were associated with social anxiety as further specification potentially could have clinical implications. We used exploratory factor analysis (EFA) [26] with SIAS, SAD, SIPS, SPRS self-consciousness and safety behaviours from t2, and all subscales of the MCQ-30 from t1 to assess which metacognition subscales loaded on the same factor as the social anxiety measures. Direct oblimin was used as the rotation method, and two factors emerged from the analysis based on the Scree plot. Factor 1 was most closely related to social anxiety as both SAD, SIAS and the self-processing variables loaded most highly. In addition, two subscales from the MCQ-30; negative metacognitive beliefs and cognitive confidence, also loaded on this factor. Positive metacognitive beliefs and cognitive self-consciousness loaded most on factor 2, so this factor appeared to be a separate metacognitive factor. The subscale of the MCQ-30; need for control, showed a co-loading on factor 1 and 2, and was therefore unclear.

**Fig 3:**
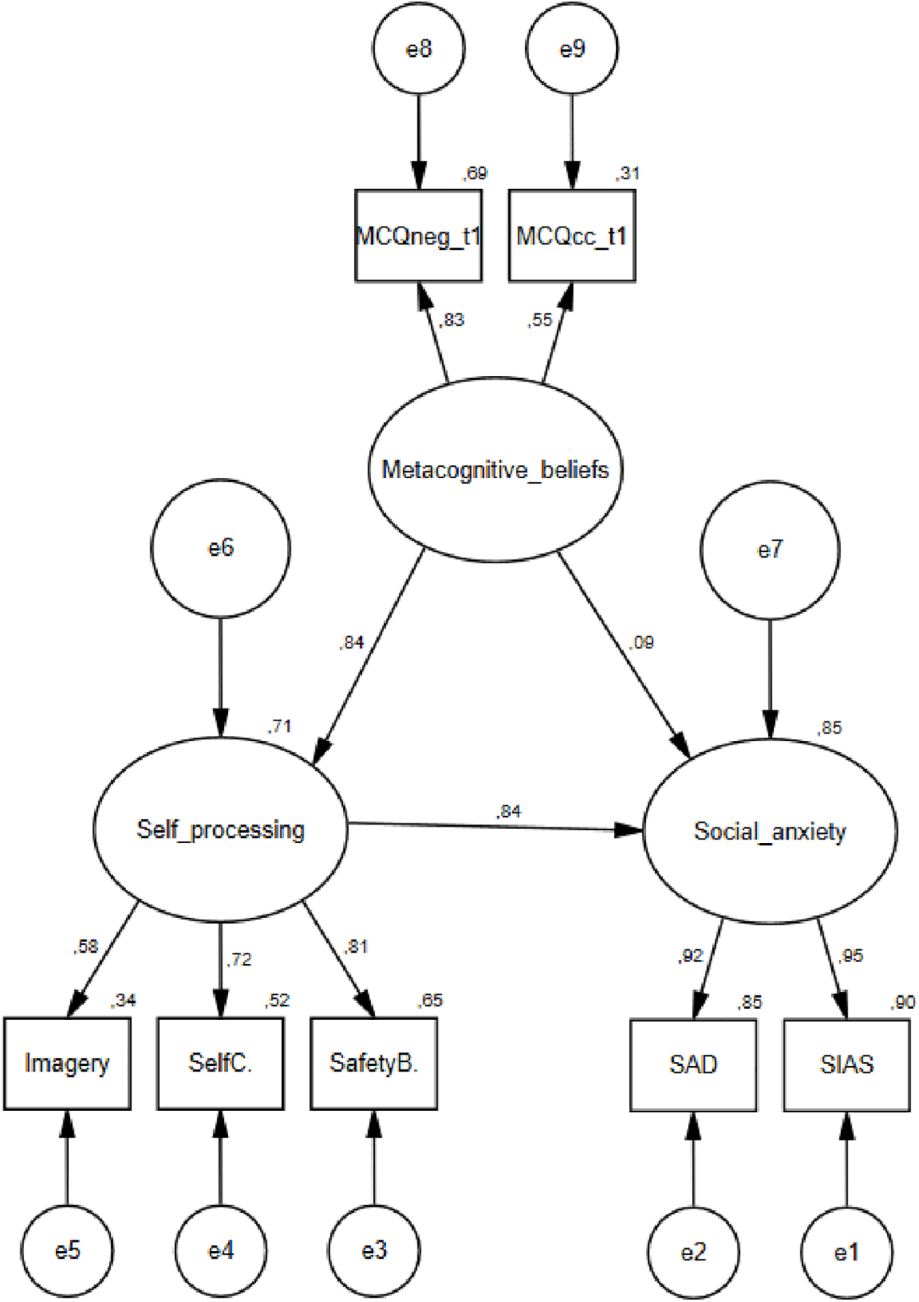
Structure and standardized estimates of the specified metacognitive model of social anxiety. MCQneg = Negative metacognitive beliefs about the uncontrollability and danger of thoughts, MCQcc = Judgements of confidence in memory.

Drawing on the EFA, we refined the metacognitive model, and created a latent metacognitive variable consisting of two observed metacognitive variables; negative metacognitive beliefs and cognitive confidence as depicted in Fig 3. We then tested the fit of this model with metacognitive beliefs from t1 specified as a latent variable and self-processing and social anxiety symptoms from t2 included as before. The fit of this model was good χ^2^(11) = 17.206, p = .102, CFI = .997, TLI = .994, RMSEA = .033, SRMR = .015, and the squared multiple correlations were .71 for self-processing, and .85 for social anxiety. All the standardized regression weights in the model except for the path from metacognitive beliefs to social anxiety was significant at the .001 level. The refined metacognitive model was not a significantly better fit than the first metacognitive model (p = .415) tested, but was a significantly better fit than the cognitive model Δ χ^2^ = -3.608, Δ df = 4, (p < .001).

## Discussion

In this study, we tested the absolute and respective fit of the cognitive and the metacognitive model of social anxiety symptoms using structural equation modelling in a longitudinal data-set. We found that the cognitive model did not fit the data well overall but inspection of a range of fit indices implied a good fit on some indices. In contrast, the metacognitive model showed a consistent and good overall fit. Furthermore, in line with the fit indices, the AIC values indicated that the metacognitive model was a better fit to the data than the cognitive model.

In an attempt to specify the metacognitive model in greater detail we ran an exploratory factor analysis to determine the latent structure among the metacognitive subscale mediators and dependent variables in the model. Two metacognitive beliefs, uncontrollability and danger of worry, and cognitive confidence loaded on the social anxiety symptom factor. We therefore used these observed variables to specify a new latent variable: ‘metacognitive beliefs’. This revised model also fitted the data well and showed an improved fit over the first metacognitive model suggesting that negative metacognitive beliefs about uncontrollability and danger of worry and beliefs concerning the effectiveness of memory may be particularly relevant for social anxiety. According to the metacognitive model [5, 12], negative metacognitive beliefs are considered to be the most important domains across disorders because these beliefs lead to the persistence of the cognitive attentional syndrome (i.e. self-processing and worry) due to a failure to attempt control and because they lead to negative and threatening interpretations of mental events. Consistent with a role of metacognitive beliefs in causing disorder a recent study by Nordahl, Nordahl, Hjemdal and Wells [17] found that improvement in SAD symptoms following treatment was predicted by change in negative metacognitive beliefs. Wells and Matthews [5] have suggested that low cognitive confidence (in memory) could be instrumental in guiding an individual to focus on internal processes in order to try to compensate. This would constitute a disposition to shift attention towards self-processing (the CAS) in social- and performance situations. Low cognitive confidence and any associated self-focus has the potential to impair social performance because the individual may withdraw effort or attention from processing the external social situation. The result of any performance impairment this might cause is likely to be the reinforcement of negative judgements of cognitive confidence and hence continued worry and self-consciousness in social situations [5].

The data add to the growing body of studies showing that metacognition is a contributor to social anxiety [15–17, 27–30]. These results may point to the need to refine existing cognitive models so that they are closer to the metacognitive model. Our findings also suggest that treatment targeting negative social beliefs (schemas) may not be necessary and that treatment should aim to formulate and modify metacognitive belief dimensions. It appears that beliefs about the uncontrollability and danger of worry and beliefs representing reduced confidence in memory may be especially relevant. Consistent with the value of moving towards more metacognitive interventions, recent treatment studies that have done so have produced very positive outcomes. In particular, these approaches have focused more on removing the CAS and reducing self-focused attention and have de-emphasised modifying social beliefs [31–33]. Moreover, change in self-focused attention and change in negative metacognitive beliefs has been found to predict symptom improvement in patients undergoing psychological treatment [17].

An incidental finding in this study was that the path from cognitive beliefs to social anxiety in the cognitive model, showed a non-significant regression weight. According to the cognitive model, cognitive beliefs, at least when expressed at the appraisal level should give rise to symptoms [2]. The failure to observe a significant path in the current model may be an effect of measuring social beliefs at an earlier distal point rather than as a more proximal maintenance process. In the specified metacognitive model, the path from metacognitive beliefs to social anxiety was also non-significant, but this is consistent with the metacognitive model where the effect of metacognitions is transmitted via the cognitive-attentional syndrome. Not with standing this, we included the direct path in the model tested here in order to hold all models constant with the aim of changing a single parameter across them; type of belief.

A limitation of our study is that our sample did not consist of patients with diagnosed social phobia. Further evaluations of the precise nature and relationships between metacognitive beliefs, the CAS (i.e. self-processing) and social anxiety in a clinical sample is required. We do not know if these models hold and the differences emerge in a clinical sample. In addition, the sample consisted predominantly of women and therefore we must be cautious in generalizing. Furthermore, controlling for time 1 social anxiety in our longitudinal models would have provided a more rigorous test on the basis of which to infer causality but it was not possible due to the high stability in the social anxiety measures (test-retest coefficient of .93 for SAD and .94 for SIAS).

In conclusion, both the cognitive and metacognitive models fitted the data well, but the metacognitive model of social anxiety was a better fit to the data than the cognitive model of social anxiety in this longitudinal data-set. Tentatively, the findings might indicate that treatment of social phobia could be advanced by addressing metacognitive beliefs, negative metacognitive beliefs about the uncontrollability and danger of thoughts and judgements of cognitive confidence in particular, rather than social-self (cognitive) beliefs. A pure metacognitive approach to treating patients with social phobia that does not focus on reality-testing the social content of cognition but instead enhances the regulation of thinking could be a beneficial alternative to CBT. These findings are important as they combine with a growing body of research showing that metacognition is a stronger determinant of psychological disorder than cognition.

## Supporting information

S1 File Social Imagery Perspective Scale (SIPS). (DOCX)

## References

[1] National Institute for Health and Care Excellence. Social anxiety disorder: recognition, assessment and treatment of social anxiety disorder. (Clinical guideline 159.) 2013. http://guidance.nice.org.uk/CG159 31869048

[2] Clark DM, Wells A. A cognitive model of social phobia In: Heimberg RG, Liebowitz MR, Hope DA, & Schneier FR. editors. Social Phobia: Diagnosis, Assessment, and Treatment. New York: The Guilford Press; 1995 p. 69–93.

[3] Rapee RM, Heimberg RG. A cognitive-behavioral model of anxiety in social phobia. Behaviour Research and Therapy. 1997;35:741–756. 9256517 10.1016/s0005-7967(97)00022-3

[4] Mayo-Wilson E, Dias S, Mavranezouli I, Kew K, Clark DM, Ades AE, et al Psychological and pharmacological interventions for social anxiety disorder in adults: a systematic review and network meta-analysis. The Lancet Psychiatry. 2014;1:368–376. doi: 10.1016/S2215-0366(14)70329-3 26361000 10.1016/S2215-0366(14)70329-3 PMC4287862

[5] Beck AT. Cognitive Therapy and the Emotional Disorders. New York: International Universities Press; 1976.

[6] Beck AT, Emery G, Greenberg RL. Anxiety disorders and phobias: A cognitive approach New York: Basic; 1985.

[7] Wells A, Matthews G. Attention and Emotion: A clinical perspective Hove UK: Erlbaum; 1994

[8] Wells A, Matthews G. Modelling cognition in emotional disorder: The S-REF model. Behaviour Research and Therapy. 1996;34:881–888. 8990539 10.1016/s0005-7967(96)00050-2

[9] Ng AS, Abbott MJ, Hunt C. The effect of self-imagery on symptoms and processes in social anxiety: a systematic review. Clinical Psychology Review. 2014;34:620–633. doi: 10.1016/j.cpr.2014.09.003 25455626 10.1016/j.cpr.2014.09.003

[10] Piccirillo ML, Dryman MT, Heimberg RG. Safety behaviors in adults with social anxiety: Review and future directions. *Be*havior Therapy. 2015;47:675–687. doi: 10.1016/j.beth.2015.11.005 27816080 10.1016/j.beth.2015.11.005

[11] Gregory B, Peters L. Changes in the self during cognitive behavioural therapy for social anxiety disorder: A systematic review. Clinical Psychology Review. 2017;52:1–18. doi: 10.1016/j.cpr.2016.11.008 27912159 10.1016/j.cpr.2016.11.008

[12] Wells A. Metacognitive therapy for anxiety and depression New York: Guilford press; 2009.

[13] Normann N, Emmerik AA, Morina N. The efficacy of Metacognitive Therapy for Anxiety and Depression: A Meta-Analytic Review. Depression and Anxiety. 2014;31:402–411. doi: 10.1002/da.22273 24756930 10.1002/da.22273

[14] Wells A. Emotional disorders and metacognition: Innovative cognitive therapy Chichester, UK: John Wiley & Sons; 2000.

[15] Nordahl HM, Nordahl H, Wells A. Metacognition and Perspective Taking Predict Negative Self-Evaluation of Social Performance in Patients with Social Anxiety Disorder. Journal of Experimental Psychopathology. 2016;7:601–607.

[16] Gkika S, Wells A. The impact of metacognitive beliefs and anticipatory processing on state anxiety in high socially anxious individuals in a speech task. Journal of Experimental Psychopathology. 2016;7:588–600.

[17] Nordahl H, Nordahl HM, Hjemdal O, Wells A. Cognitive and metacognitive predictors of symptom improvement following treatment for social anxiety disorder. Clin Psychol Psychother. 2017:1–7. 28295802 10.1002/cpp.2083

[18] Mattick RP, Clarke JC. Development and validation of measures of social phobia scrutiny fear and social interaction anxiety. Behaviour Research and Therapy. 1998;36:455–470. 9670605 10.1016/s0005-7967(97)10031-6

[19] Watson D, Friend R. Measurement of social-evaluative anxiety. Journal of Consulting and Clinical Psychology. 1969;33:448–457. 5810590 10.1037/h0027806

[20] Wells A. Cognitive Therapy of Anxiety Disorders: A practice manual and conceptual guide. John Wiley & Sons; 1997.

[21] Wells A, Cartwright-Hatton S. A short form of the metacognitions questionnaire: properties of the MCQ-30. Behaviour Research and Therapy. 2004;42:385–396. doi: 10.1016/S0005-7967(03)00147-5 14998733 10.1016/S0005-7967(03)00147-5

[22] Bentler PM. EQS structural equations program manual. Multivariate software. 1995.

[23] Hu LT, Bentler PM. Cutoff criteria for fit indexes in covariance structure analysis: Conventional criteria versus new alternatives. Struct. Equ. Model. 1999;6:1–55.

[24] Akaike H. A new look at the statistical model identification. IEEE transactions on automatic control. 1974;19:716–723.

[25] Akaike H. Factor analysis and AIC. Psychometrika. 1987;52:317–332.

[26] Kline P. An easy guide to factor analysis Routledge: 1994.

[27] Fisak B, Hammond AN. Are positive beliefs about post-event processing related to social anxiety?. Behaviour Change. 2013;30:36–47.

[28] Wong QJ, Moulds ML. Do socially anxious individuals hold positive metacognitive beliefs about rumination?. Behaviour Change. 2010; 27:69–83.

[29] Vassilopoulos SP, Brouzos A, Moberly NJ. The Relationships Between Metacognition, Anticipatory Processing, and Social Anxiety. Behaviour Change. 2015;32:114–126.

[30] Dannahy L. Stopa L. Post-event processing in social anxiety. Behaviour Research and Therapy. 2007;45:1207–1219. doi: 10.1016/j.brat.2006.08.017 17112463 10.1016/j.brat.2006.08.017

[31] Wells A, Papageorgiou C. Brief cognitive therapy for social phobia: a case series. Behaviour Research and Therapy. 2001;39:713–720. 11400714 10.1016/s0005-7967(00)00036-x

[32] Nordahl HM, Vogel PA, Morken G, Stiles TC, Sandvik P, Wells A. Paroxetine, Cognitive Therapy, and their Combination in the Treatment of Social Anxiety Disorder with and without Avoidant Personality Disorder: A Randomized Controlled Trial. Psychotherapy and Psychosomatics. 2016;85:346–356. doi: 10.1159/000447013 27744447 10.1159/000447013

[33] Vogel PA, Hagen R, Hjemdal O, Solem S, Smeby MCB, Strand ER, et al Metacognitive Therapy Applications in Social Phobia: An Exploratory Study of the Individual and Combined Effects of the Attention Training Technique and Situational Attentional Refocusing. Journal of Experimental Psychopathology. 2016;7:608–618.

