# Supplementary material for: Testing the metacognitive model against the benchmark CBT model of social anxiety disorder: Is it time to move beyond cognition?
Source: PLoS One. 2017 May 4;12(5):e0177109. doi: 10.1371/journal.pone.0177109 (PMC5417561; doi:10.1371/journal.pone.0177109)
Supplement: S1 File — (DOCX) [file pone.0177109.s001.docx]

**Social Imagery Perspective Scale (SIPS)**

Below are items that assess mental images people have in social situations. Please read each one and indicate how often you have these experiences.

| When I’m in a social situation: | | Never | Sometimes | Often | Almost always |
| --- | --- | --- | --- | --- | --- |
| 1. | I have an image of how I appear to other people | 0 | 1 | 2 | 3 |
| 2. | I see myself as if from someone else’s point of view | 0 | 1 | 2 | 3 |
| 3. | I have an inner impression of how I look | 0 | 1 | 2 | 3 |

**Norwegian:**

**Sosialt forestillingsbilde-perspektiv skala (SIPS)**

Under er ledd som måler mentale bilder folk har i sosiale situasjoner. Vennligst les hver enkelt og indiker hvor ofte du har slike opplevelser.

| Når jeg er i en sosial situasjon: | | Aldri | Noen ganger | Ofte | Nesten alltid |
| --- | --- | --- | --- | --- | --- |
| 1. | Har jeg et bilde av hvordan jeg fremstår for andre folk | 0 | 1 | 2 | 3 |
| 2. | Ser jeg meg selv som fra noen andres synspunkt | 0 | 1 | 2 | 3 |
| 3. | Har jeg et indre inntrykk av hvordan jeg ser ut | 0 | 1 | 2 | 3 |
